# Time-keeping and decision-making in living cells: Part II

**DOI:** 10.1098/rsfs.2022.0024

**Published:** 2022-06-10

**Authors:** John J. Tyson, Attila Csikasz-Nagy, Didier Gonze, Jae Kyoung Kim, Silvia Santos, Jana Wolf

**Affiliations:** ^1^ Department of Biological Sciences, Virginia Polytechnic Institute & State University, Blacksburg, VA 24061, USA; ^2^ Faculty of Information Technology and Bionics, Pázmány Péter Catholic University, 1088 Budapest, Hungary; ^3^ Unit of Theoretical Chronobiology, Université Libre de Bruxelles, 1050 Brussels, Belgium; ^4^ Department of Mathematical Sciences, KAIST, Daejeon 34141, South Korea; ^5^ Biomedical Mathematics Group, Institute for Basic Science, Daejeon 34126, South Korea; ^6^ Quantitative Stem Cell Biology Laboratory, The Francis Crick Institute, London NW1 1AT, UK; ^7^ Mathematical Modeling of Cellular Processes, Max Delbrück Center for Molecular Medicine, 13125 Berlin, Germany; ^8^ Department of Mathematics and Computer Science, Free University, 14195 Berlin, Germany

**Keywords:** molecular regulatory networks, mathematical modelling, development, cell cycle, differentiation, cellular heterogeneity

## 1. Introduction

This two-part collection of articles on ‘time-keeping and decision-making in living cells' covers various examples of the mechanisms by which cells and organisms receive and integrate signals from many sources, figure out how the organism should respond and then elicit the appropriate response. Unlike digital computers, these information-processing systems (IPS) are autonomous, analogue and massively parallel, and their responses are remarkably successful in supporting the survival, growth, repair and reproduction of living organisms. Molecular, cellular and organismal biologists, in collaboration with mathematical and computational biologists, have made remarkable progress in understanding how living IPS work. Some of these recent successes are reviewed in this collection.

Part I focused on time-keeping, in particular on mechanisms of biological oscillators, on synchronization of intercommunicating oscillators and on entrainment to external driving rhythms, with particular emphasis on circadian rhythms. Jimenez *et al.* [1] provided a valuable survey of entrainment among biological oscillators, focusing on the circadian clock, the cell cycle, cardiac pacemaker cells, glycolytic oscillations and inflammatory responses. Goldbeter & Yan [2] presented a masterly review of multi-rhythmicity (two or more simultaneously stable oscillatory states) and multi-synchronization (two or more simultaneously stable modes of synchronization), with examples drawn from cyclic AMP signalling, circadian rhythms and cell cycle oscillations. Burckard *et al.* [3] provided new results on the synchronization of peripheral circadian clocks by intercellular communication between two cells or in small clusters of cells. And Jeong *et al.* [4] investigated the role of multiple modes of transcriptional repression in generating many of these rhythms.

**Figure 1.**
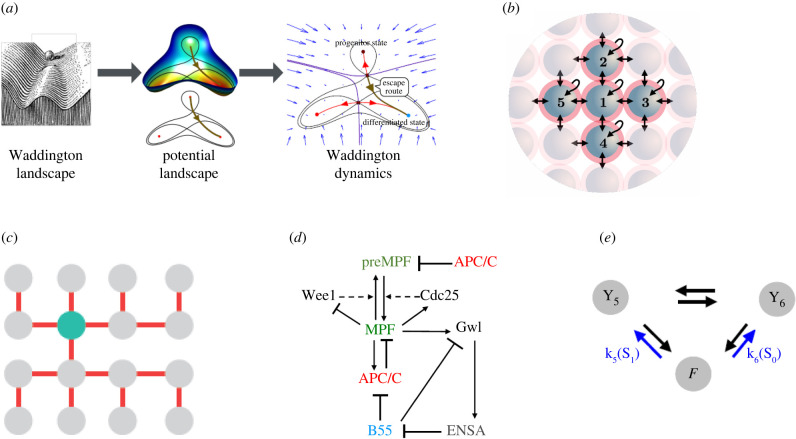
Cell fate decisions. (*a*) From Waddington's landscape to Waddington dynamics; from Sáez *et al.* [5]. Flow lines, which are not necessarily perpendicular to the potential contour lines, describe cell differentiation dynamics. (*b*) Multi-cellular model for cell specification in an early mammalian embryo into epiblast (expressing Nanog) and primitive endoderm (expressing Gata6); from Robert *et al.* [6]. Each cell synthesizes Fgf4 at a rate proportional to its level of Nanog. In response to the average level of Fgf4 in its neighbourhood, each cell upregulates Gata6 and downregulates Nanog. (*c*) A 16-cell germline cyst in *Drosophila melanogaster*; from Diegmiller *et al.* [7]. The oocyte (green ball) is supported by nurse cells (grey balls), which connect to the oocyte by intercellular bridges (red lines). (*d*) The protein interaction network controlling progression through the eukaryotic cell cycle; from Tyson & Novak [8]. MPF = M-phase promoting factor (a CDK); APC/C = anaphase promoting complex; Wee1 and Gwl are protein kinases, Cdc25 and B55 are protein phosphatases; ENSA is a stoichiometric inhibitor. (*e*) A linear framework graph for an enzyme-catalysed reaction; from Nam *et al.* [9]. The phosphatase F removes a phosphate group from S_1_, via the complexes Y_5_ = F : S_1_ and Y_6_ = F : S_0_, to release the unphosphorylated form S_0_. The edges represent the biochemical reactions and the labels denote the reaction rates. In principle, all reactions are reversible, although, in a living cell, the reaction Y_6_ → Y_5_ is much slower than Y_5_ → Y_6_. All figures used by permission of the authors and the publisher.

Part II focuses on decision-making in cell differentiation, development and cell cycle progression. Sáez *et al.* [5] use ideas from dynamical systems theory to turn Waddington's ‘landscape’ metaphor of cell fate decisions into quantitative and predictive models that can shed light on the underlying biology (figure 1*a*). For instance, they identify just two distinct ways for a cell to choose between alternate fates: the ‘binary choice’ landscape and the ‘binary flip’ landscape. They go on to study three-attractor landscapes and beyond, and to consider how to incorporate experimental data with dynamics. Robert *et al.* [6] investigate potential sources of heterogeneity required to induce cell differentiation in early mammalian embryos, using a multi-cellular model of Nanog-Gata6-Fgf4 interactions (figure 1*b*). They attribute the observed characteristics to cell-to-cell variability in gene expression, most notably Nanog expression. Diegmiller *et al.* [7] study the dynamics of cell division and differentiation in small clusters of cells that make up germline cysts, which ultimately mature into an oocyte and surrounding support cells (figure 1*c*). They propose a minimal cell cycle oscillator model for generating the cell lineage tree (CLT) of a cyst and discuss how CLTs of varying topologies can arise. Such clonal clusters of connected cells are found in almost all lineages of eukaryotes, and the cytoplasmic bridges that connect such cells are thought to have played a key role in the evolution of multi-cellularity. Tyson & Novak [8] use mathematical models to study the roles of time-keeping and decision-making during progression through the eukaryotic cell cycle. Their models, based on the well-known cyclin-dependent kinase (CDK) control system (figure 1*d*), account for both clock-like CDK oscillations during early embryonic cell divisions and switch-like CDK-arrested states (checkpoints) during somatic cell cycles. Lastly, Nam *et al.* [9] review a graph-based approach to biochemical reaction dynamics, called ‘the linear framework’ (figure 1*e*). In this approach, the nonlinear kinetics of a network of biochemical reactions is decomposed into a coupled set of graphs, each of which has linear dynamics, and the steady states of the network can be expressed as rational algebraic functions of the parameters. The linear framework, which encompasses systems both at thermodynamic equilibrium and away from equilibrium, provides a sound theoretical foundation for modelling the post-translational modifications that underlie many biochemical mechanisms of time-keeping and decision-making in living cells.

## 2. Decision-making in cell physiology

### 2.1. Early studies

**Figure 2.**
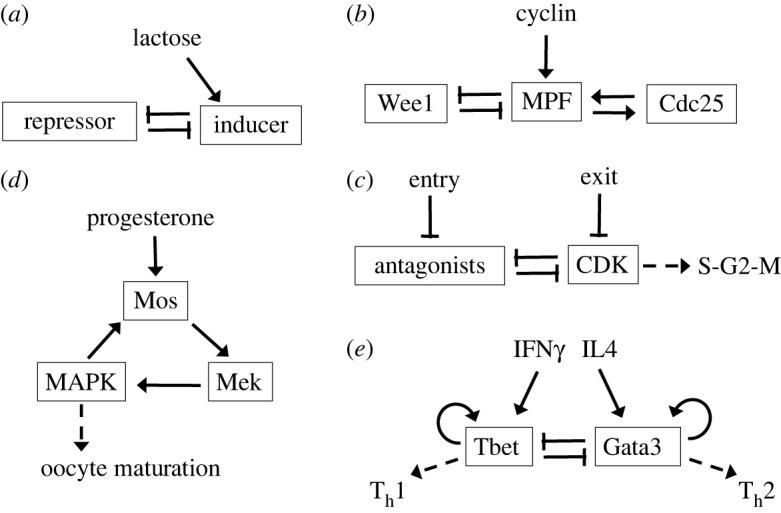
Decision-making by bistable molecular circuits. (*a*) The *lac* operon. The *lac* repressor blocks transcription of lactose-metabolizing proteins. If lactose is present in the growth medium (and glucose is absent), lactose enters the cell and isomerizes to allolactose, which binds to and inactivates the repressor. This net-positive feedback loop (+−−) is the basis of ‘all-or-none’ behaviour of the *lac* operon. (*b*) Mitotic entry. The activity of M-phase promoting factor (MPF ) is inhibited by Wee1 and activated by Cdc25. Cyclin synthesis drives the accumulation of MPF molecules, which abruptly switch from the inactive to active form. The switch exhibits bistability, a consequence of the (−−) and (++) feedback loops. (*c*) Control of progression through the eukaryotic cell cycle. CDKs, which drive DNA synthesis and mitosis, are opposed by ‘antagonists,’ including stoichiometric inhibitors and cyclin-degrading pathways. Mutual inhibition (−−) creates a bistable switch between a phase of low CDK activity (G1) and high activity (S-G2-M). The G1-to-S transition is triggered by ‘entry’ proteins that downregulate the antagonists, and the M-to-G1 transition by ‘exit’ proteins that downregulate CDK activity. (*d*) Oocyte maturation. In frog ovaries, progesterone stimulates a cohort of immature oocytes into a state receptive to fertilization. Maturation is initiated by MAP kinase (MAPK) activation, which is a bistable response to progesterone created by the positive feedback (+++) loop. (*e*) T-helper cell differentiation. The interactions of the transcription factors Tbet and Gata3 (which control the differentiation of T-helper 1 and T-helper 2 cells, respectively) constitute a MISA motif (mutual inhibition self-activation). The outcome of the interactions (there are four possible stable steady states) is controlled by the cytokines interferon-gamma and interleukin-4.

Experimental and theoretical studies of decision-making by bistable molecular circuits go back many years, at least to the observations of Novick & Weiner [10] on the ‘all-or-none’ behaviour of the *lac* operon (figure 2*a*) and later mathematical models by Griffith [11], Thomas [12] and Santillán & Mackey [13]. In cell cycle studies, Solomon *et al.* [14] observed an abrupt activation of CDK activity, which was later attributed to bistability in a mathematical model of the feedback control of CDK (figure 2*b*) [15], and bistable behaviour was demonstrated experimentally by Sha *et al.* [16] and Pomerening *et al.* [17]. Nasmyth [18] proposed that—quite generally—progression through the eukaryotic cell cycle involves irreversible switching between two ‘self-maintaining’ states: low CDK activity (G1 phase) and high CDK activity (S-G2-M phases). The origin of this behaviour is the mutual antagonism between CDKs and their ‘enemies’ (stoichiometric inhibitors and cyclin-degrading pathways; figure 2*c*), as made clear later by mathematical modelling [19]. Ferrell & Machleder [20] observed an ‘all-or-none’ maturation response of frog oocytes to progesterone treatment, which they attributed to bistability in the MAP kinase signalling pathway (figure 2*d*). Yates *et al.* [21] and later van den Ham & de Boer [22] studied the phenotypic polarization of helper T cells with nonlinear differential equations based on the regulatory properties of master transcription factors (mutual inhibition and self-activation (MISA); figure 2*e*). van den Ham & de Boer found up to four stable steady states: naive cell (both factors off), Th1 cell (Tbet on), Th2 cell (Gata3 on) and dual-expressing cell (both factors on). The same motif was introduced by Huang *et al.* [23] to model the differentiation of blood cell progenitors into erythroid and myeloid cell lineages. Chikarmane *et al.* [24] modelled the differentiation of embryonic stem cells in terms of two basic transcription factors (Oct4-Sox2 dimer and Nanog) that mutually activate each other, creating a bistable switch with the transcription factors being either on or off. Perkins & Swain [25] and Balázsi *et al.* [26] have reviewed optimal decision-making in noisy environments.

The common themes of these early studies are that (i) cells make decisions by flipping between coexisting stable steady states (bistability or multi-stability) and (ii) multiple stable steady states are generated by biochemical reaction networks with mutual inhibition and/or self-activation [27]. We see these themes repeated over and over again in more recent developments, with interesting twists.

### 2.2. Bistability and multi-stability in models of stem cell differentiation

**Figure 3.**
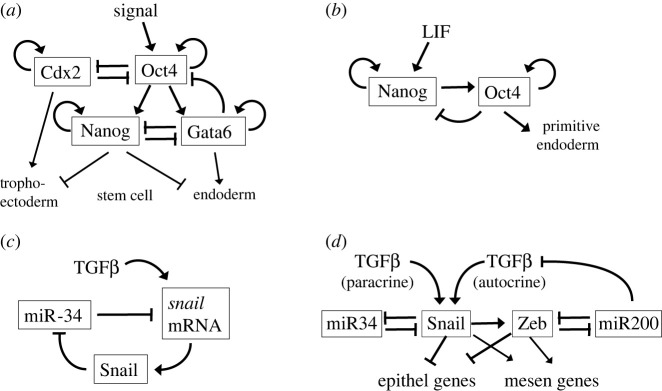
Differentiation of stem cells. (*a*) The differentiation of stem cells into trophectoderm or endoderm is controlled by two interacting MISA motifs. The outcome (one of four possible stable steady states) is determined by the strength of the input signal and, of course, the strengths of the interactions within the network. (*b*) A simplified view of the network in (*a*). Nanog activates Oct4 indirectly by inhibiting Gata6. Oct4, at high concentration, inhibits Nanog indirectly by activating Gata6. This ‘activator–inhibitor’ motif has a single, stable, excitable steady state of high Nanog activity. Random fluctuations may kick the cell off the steady state and into a transient region of low Nanog activity, when the cell is prone to differentiate into primitive endoderm. The signal is LIF (leukaemia inhibiting factor). (*c*) Mutual inhibition between *snail* mRNA and its antisense microRNA (miR-34). The double-negative feedback loop generates bistability, controlled by the signal from transforming growth factor-beta (TGFβ). (*d*) A model of the EMT. EMT is controlled by two transcription factors, Snail and Zeb, which both exhibit bistable responses to TGFβ because their synthesis is controlled by microRNAs (miR34 and miR200) as in (*c*).

The differentiation of pluripotent embryonic stem cells and of blood cell progenitors has long fascinated mathematical biologists (e.g. an early review by Laurent & Kellersohn [28]). MISA motifs are hallmarks of the study by Chickarmane & Peterson [29] on the differentiation of stem cell, trophoectoderm and endoderm lineages; figure 3*a*. They found that, as the signal is varied, the control system may exhibit four different steady states: trophoectoderm, endoderm, stem cell and ‘differentiated’ stem cell. Later, Chickarmane *et al.* [30] used a similar model to study the role that stochastic noise in gene expression plays in the differentiation process, concluding that Nanog heterogeneity is the deciding factor in stem cell fate. In the meantime, in a study of Nanog expression in mouse embryonic stem cells, Kalmar *et al.* [31] observed two populations of cells: HN cells (high Nanog, relatively stable) and LN cells (low Nanog, relatively unstable but more likely to differentiate). To account for their observations, they proposed a model of noise-driven excitability (figure 3*b*; an activator–inhibitor motif rather than a MISA motif).

Mutual inhibition between mRNA and microRNA has been proposed by Tian *et al.* [32] as a mechanism for bistable switches in cell fate decisions (figure 3*c*). They applied this idea to the epithelial-to-mesenchymal transition (EMT) in embryonic development [33] with a mathematical model based on two bistable switches (figure 3*d*) controlling the expression of the transcription factors Snail and Zeb. At low TGFβ (the inducer of EMT), Snail and Zeb are turned off, and the cell is expressing epithelial genes. At intermediate TGFβ, Snail and Zeb are turned on partially, and the cell is not secreting TGFβ (partial EMT state). At higher levels of (paracrine) TGFβ, the cell turns on Snail and Zeb fully and starts secreting TGFβ, which locks the cell in the mesenchymal state by autocrine signalling, even if the external TGFβ signal drops substantially.

The early work of Yates *et al.* [21], Huang *et al.* [23] and van den Ham & de Boer [22] was followed up by Huang [34] and Hong *et al.* [35–37], who used MISA motif models to understand in more detail the differentiation of CD4+ T cells into multiple lineages and into hybrid cells expressing multiple cytokines.

### 2.3. Multi-cell/multi-scale modelling

Up to this point, we have attributed cell differentiation to bistability and multi-stability at the single-cell level, based on MISA motifs in the underlying control circuits. In a fascinating paper, Stanoev *et al.* [38] recently proposed a conceptually different dynamical mechanism in which cell types emerge and are maintained collectively by cell–cell communication as a novel inhomogeneous state of the coupled system. They showed how spatial patterns of cell differentiation (inhomogeneous steady states) arise in a population of cells as cell number increases. Robust proportions of differentiated cells emerge spontaneously and recover reliably after perturbations.

De Mot *et al.* [39] proposed a multi-cellular model for the differentiation of the inner cell mass into epiblast and primitive endoderm based on tristability in the Nanog-Gata6-Fgf4 network (figure 1*b*). The model was later extended to account for cell division [40]. Saiz *et al.* [41] proposed a related model that further highlights the role of Fgf4 signalling between cells in the cell fate decision and in the embryo's response to perturbations in lineage composition.

An important aspect of embryonic development is the coupling of mechanics to gene expression in the context of cellular tissue that is increasing in cell number due to division. Extending their model of Cdx2/Oct4 and Nanog/Gata3 interactions (figure 3*a*) to include mechanical forces and cell division, Krupinski *et al.* [42] have attacked this problem in the context of pattern formation in the mammalian blastocyst. Their multi-cell/multi-scale approach is a powerful tool to model how cells move within the embryo in response to mechanical forces and how cell positions influence cell fates (the formation of trophectoderm and of endoderm).

Macklin *et al.* [43] presented a multi-scale model of solid tumour growth, which illustrated the potential of mathematical modelling to understand complex interactions of cell proliferation, extracellular matrix degradation, angiogenesis and nutrient availability on the ability of solid tumours to expand. Populations of budding yeast cells growing on sucrose were studied by Dai *et al.* [44]. The population was maintained by daily dilution with fresh medium. Because sucrose is hydrolysed to glucose and fructose externally, the cells benefit from neighbours (called the Allee effect in population biology), and if the daily dilution factor is too large, the population undergoes a catastrophic collapse from a stable population density to extinction. The ‘tipping point’ is a saddle-node bifurcation, and close to this point the population should become more vulnerable to fluctuations (loss of resilience), which they demonstrated experimentally. In bacterial biofilms, a different type of vulnerability arises from a conflict between interior and peripheral cells. Peripheral cells protect interior cells from chemical attack but, at the same time, starve interior cells of nutrients. Liu *et al.* [45] showed that biofilm cells resolve this conflict by periodically halting growth to increase nutrient availability to interior cells. The oscillations, observed period 2–3 h, arise by Hopf bifurcations in the mathematical model.

### 2.4. Cell cycle modelling: checkpoints, variability and travelling waves

Cell cycle modelling has moved in several directions over the past decade. Regarding mammalian cell cycle regulation, Gérard & Goldbeter [46,47] presented a limit-cycle model of the CDK control system and assessed its merits. Weis *et al.* [48] confronted a published bistable switch model of CDK controls [49,50] with novel quantitative data on cyclin A2 and cyclin B1 accumulation, which suggested some modifications to the published model. Heldt *et al.* [51] presented a model of light-responsive size control of DNA replication in *Chlamydomonas*, to explain the unusual ‘multiple-fission’ mode of cell division in this green alga. Li *et al.* [52] presented an elaborate, stochastic, spatio-temporal model of the asymmetric cell division cycle of *Caulobacter crescentus*, an alphaproteobacterium. Deterministic modelling of the budding yeast cell cycle has become more comprehensive. Palumbo *et al.* [53] presented a detailed model of the G1–S transition, based on multi-site phosphorylation of Whi5, an inhibitor of transcription of cyclin genes. Kraikivski *et al.* [54] presented a model of the budding yeast cell cycle (from start to finish) that tracks the dynamics of approximately 60 molecular species by a set of differential-algebraic equations. The model was fitted to the observed phenotypes of 263 mutant strains of budding yeast with 98% success (six strains could not be correctly simulated). Stochastic modelling has also progressed; the latest model by Barik *et al.* [55], which follows the expression of 17 genes (mRNAs and proteins) by Gillespie's stochastic simulation algorithm, provides excellent quantitative fit to observed measurements of cell cycle variability in wild-type cells and approximately 20 mutant strains. In particular, the model accounts for ‘partial viability’ of some mutant strains, which is a property that cannot be explained by a deterministic model. Other recent papers have explored the roles of checkpoints in the cell cycle, for example, the DNA damage checkpoint [56–58], the spindle assembly checkpoint [59], mitotic entry and exit [60] and the restriction point [61]. Comprehensive Boolean (discrete logical) models of the budding yeast cell cycle have also been proposed, starting with Fauré *et al.* [62] and pursued subsequently by Münzner *et al.* [63] at a ‘genome scale’ and by Howell *et al.* [64] to incorporate spatial effects into a logical model of mitotic exit.

Because the activation of MPF (a cyclin-CDK dimer) is a bistable switch (figure 2*b*), Novak & Tyson [65] predicted that ‘trigger’ waves of MPF activation would propagate in syncytial (i.e. multi-nucleate) tissues at a speed of 10–100 µm min^−1^. Twenty years later, these waves were observed definitively by Chang & Ferrell [66] in frog egg extracts supplemented with sperm nuclei. The waves travelled at approximately 50 µm min^−1^.

### 2.5. Synthetic regulatory circuits

The age of synthetic genetic regulatory networks was inaugurated by the ground-breaking papers of Gardner *et al.* [67]—the genetic toggle switch, and Elowitz & Leibler [68]—the repressilator. Stricker *et al.* [69] created the first robust, tunable, synthetic gene oscillator, based on an activator–inhibitor motif (like figure 3*b*), in *E. coli* cells. Tigges *et al.* [70] created a tunable synthetic oscillator in a mammalian cell with a transcriptional control circuit encoding both positive and time-delayed negative feedback loops. Danino *et al.* [71] showed that a population of oscillating cells could be synchronized by global intercellular coupling, which was introduced by cloning the *Vibrio* quorum sensing machinery into their oscillating *E. coli* strain. Zhang *et al.* [72] designed and implemented a synthetic NF-κB oscillator in budding yeast cells, based on RelA (a nuclear factor *κ*B protein) and I*κ*B*α* (an inhibitor of RelA).

Matsuda *et al.* [73] studied ‘cell-type bifurcation’ of Chinese hamster ovary cells that were engineered with a basic transcriptional repression circuit based on Delta-Notch signalling between cells supplemented with an intracellular self-activation circuit whereby Notch induces expression of Lfng (Lunatic Fringe) and Lfng activates Notch. The population consisted of a mixture of Delta-expressing cells (low Notch and Lfng) and Lfng-expressing cells (low Delta and high Notch).

Sekine *et al.* [74] have engineered a reaction­–diffusion patterning network in human embryonic kidney cells using the Nodal–Lefty signalling system, which satisfies—in principle—the requirements of Turing pattern formation: Nodal activates the production of both Nodal and Lefty, Lefty inhibits the activity of Nodal, and the diffusion range of Lefty (the inhibitor) is approximately 3.5 times longer than Nodal (the activator). Nonetheless, the authors propose that the patterns they observe are not Turing patterns but ‘solitary localized structures' caused by an excitable or bistable reaction–diffusion system with a rapidly diffusing inhibitor. In this mechanism, Nodal foci are formed by short-range self-activation and further propagation of Nodal activation is stopped by long-range inhibition.

The potential for synthetic decision-making has been greatly expanded by two publications. Gordley *et al.* [75] showed how slow-acting, synthetic bistable switches (in yeast cells) can be selectively tuned by fast-acting, synthetic phospho-regulons. Zhu *et al.* [76] introduced ‘MultiFate’ technology for creating synthetic circuits that support controllable and expandable multi-stability in mammalian cells. MultiFate circuits are created from synthetic zinc-finger transcription factors that enable homodimer-dependent self-activation and heterodimer-dependent cross-inhibition. The MultiFate-2 circuit is the MISA motif introduced in figure 2*e*; it readily generates bistability and tristability in a controllable fashion. MultiFate-3 cells can generate up to seven stable steady states.

### 2.6. Pattern formation in bistable systems

Shortly after fertilization, the *C. elegans* zygote establishes an anterior–posterior gradient of PAR proteins in the cell cortex. In modelling this phenomenon, Goehring *et al.* [77] found that passive advection of PAR proteins by transient actomyosin-driven flow in the cell cortex can serve as a mechanical trigger for the formation of a persistent spatial pattern in a reaction–diffusion system exhibiting bistability. Bistability in their model is generated by mutually antagonistic interactions between ‘anterior’ and ‘posterior’ PAR proteins.

All above-ground plant tissues originate from stem cell divisions in shoot apical meristems (SAM). Stem cells, in the central zone of SAM, express the transcription factor WUSCHELL, which upregulates its own inhibitors, encoded by *CLAVATA* genes. Spontaneous emergence of a central zone is often modelled by a Turing mechanism, for example [78], but Battogtokh [79] has shown that pattern formation in a bistable system gives a better description of the nucleation and confinement of the stem cell domain. His proposal for SAM patterning closely resembles patterning in the Nodal–Lefty system developed by Sekine *et al*. [74].

### 2.7. Programmed cell death

Apoptosis is an interesting cell fate decision whereby, in response to severe stress, a cell commits ‘suicide’. Crucially, this decision, once made, must be irreversible, and a one-way bistable switch is ideally suited to this end. Following on early models of irreversible apoptosis in mammalian cells [80–83], Ziraldo & Ma [84] presented a mathematical model of the apoptotic switch in the fruit fly and discussed the role of feedback topology on the reversibility or irreversibility of the switch. Autophagy (self-feeding) is another interesting cellular stress response, whereby a cell breaks down its own macromolecules to obtain energy and raw materials for survival purposes. By design, autophagy is reversible, so that the cell can recover if the stress is removed soon enough. If the stress is too intense, autophagy (which can be lethal if taken too far) is usually coordinated with the intrinsic apoptotic death pathway. Kapuy *et al.* [85] have modelled this crosstalk between apoptosis and autophagy and the positive feedback loop that makes the apoptosis switch irreversible.

## 3. Conclusion

Altogether, the studies reviewed in Parts I and II of this Special Issue have contributed greatly to our understanding of the molecular mechanisms underlying biological information processing, giving us a deeper appreciation of the—often non-intuitive—dynamical interplay of biochemical switches and clocks and the life-sustaining processes that they support. The progress resulting from the development, analysis and application of mathematical models has revolutionized our interpretation of experimental observations and renewed our vision of future possibilities in health science and biotechnology.

## Data Availability

This article has no additional data.
